# The *C*. *elegans* Discoidin Domain Receptor DDR-2 Modulates the Met-like RTK–JNK Signaling Pathway in Axon Regeneration

**DOI:** 10.1371/journal.pgen.1006475

**Published:** 2016-12-16

**Authors:** Naoki Hisamoto, Yuki Nagamori, Tatsuhiro Shimizu, Strahil I. Pastuhov, Kunihiro Matsumoto

**Affiliations:** Division of Biological Science, Graduate School of Science, Nagoya University, Chikusa-ku, Nagoya, Japan; University of California San Francisco, UNITED STATES

## Abstract

The ability of specific neurons to regenerate their axons after injury is governed by cell-intrinsic regeneration pathways. However, the signaling pathways that orchestrate axon regeneration are not well understood. In *Caenorhabditis elegans*, initiation of axon regeneration is positively regulated by SVH-2 Met-like growth factor receptor tyrosine kinase (RTK) signaling through the JNK MAPK pathway. Here we show that SVH-4/DDR-2, an RTK containing a discoidin domain that is activated by collagen, and EMB-9 collagen type IV regulate the regeneration of neurons following axon injury. The scaffold protein SHC-1 interacts with both DDR-2 and SVH-2. Furthermore, we demonstrate that overexpression of *svh-2* and *shc-1* suppresses the delay in axon regeneration observed in *ddr-2* mutants, suggesting that DDR-2 functions upstream of SVH-2 and SHC-1. These results suggest that DDR-2 modulates the SVH-2–JNK pathway via SHC-1. We thus identify two different RTK signaling networks that play coordinated roles in the regulation of axonal regeneration.

## Author Summary

An axon’s ability to regenerate after injury is governed by cell-intrinsic regeneration pathways. The *C*. *elegans* JNK MAP kinase pathway is required for the regrowth of neurons after injury. Previously, we identified several *svh* genes involved in JNK-mediated signaling. Among them, the *svh-1* and *svh-2* genes encode a growth factor and its receptor tyrosine kinase (RTK), respectively. This SVH-1–SVH-2 signaling cascade positively regulates axon regeneration through the JNK pathway. In the present study, we investigate the role of the *svh-4*/*ddr-2* gene, which encodes an RTK containing a discoidin domain that is activated by collagen. Indeed, DDR-2 functions downstream of EMB-9 collagen type IV. Here, we show that the *ddr-2* and *emb-9* mutations delay initiation of regeneration after axon injury. Furthermore, we demonstrate that DDR-2 modulates the SVH-1–SVH-2–JNK pathway through the scaffold protein SHC-1. Thus, two different RTK signaling networks play coordinated roles in the regulation of axonal regeneration.

## Introduction

The ability of a neuron to regenerate in response to injury is modulated by a balance of extrinsic factors that promote or inhibit axon outgrowth, and by intrinsic processes that regulate axon growth potential. Most invertebrate neurons are able to regenerate, as are neurons in the mammalian peripheral nervous system. By contrast, neurons in the mammalian central nervous system have limited regenerative capability [1]. Although both extrinsic and intrinsic regeneration signals can influence regenerative success, the specific signaling pathways underlying neuronal regeneration have yet to be fully elucidated.

**Fig 1:**
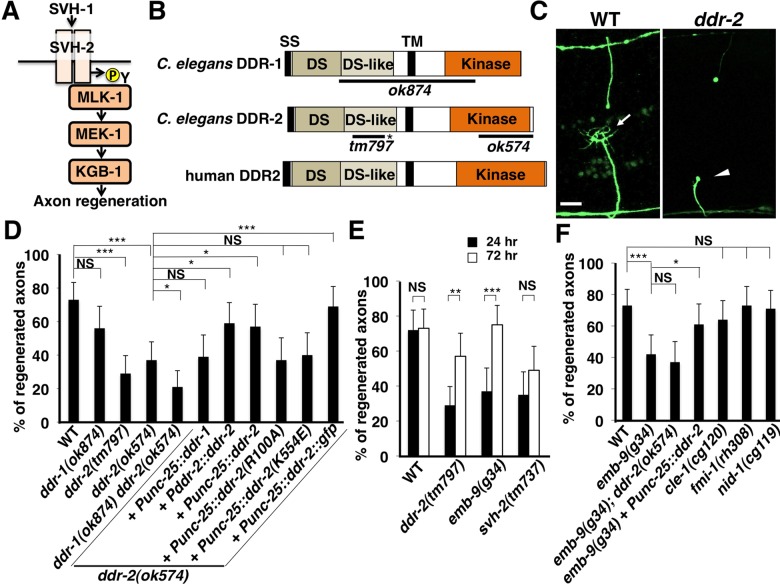
DDR-2 is required for efficient axon regeneration in *C*. *elegans*. (A) SVH-2–JNK MAPK pathway required for axon regeneration in *C*. *elegans*. The growth factor SVH-1 and its receptor tyrosine kinase SVH-2 promote axon regeneration through tyrosine phosphorylation of MLK-1 in the KGB-1 JNK pathway. (B) Structures of DDR-1 and DDR-2. Schematic diagrams of DDR-1, DDR-2 and their mammalian counterpart DDR2 are shown. Domains are shown as follows: a signal sequence (SS), a discoidin domain (DS), a DS-like domain, a transmembrane domain (TM), and a tyrosine kinase domain (Kinase). The bold lines underneath indicate the extent of the deleted region in each deletion mutant. The *ddr-2(tm797)* mutation causes a premature translation stop (indicated by asterisk) in the extracellular domain. (C) Representative D-type motor neurons in wild-type and *ddr-2* mutant animals 24 hr after laser surgery. In wild-type animals, a severed axon has regenerated a growth cone (arrow). In *ddr-2* mutants, proximal ends of axons failed to regenerate (arrowheads). Scale bar = 10 μm. (D,F) Percentages of axons that initiated regeneration 24 hr after laser surgery. Error bars indicate 95% CI. *P<0.05, ***P<0.001. NS, not significant. (E) Percentages of axons that initiated regeneration 24 or 72 hr after laser surgery. Error bars indicate 95% CI. **P<0.01, ***P<0.001. NS, not significant.

The nematode *Caenorhabditis elegans* has been successfully used as a model system to study the mechanisms of axon regeneration [2,3]. Its amenability to genetic manipulation makes it particularly useful for uncovering novel factors involved in the regulation of this axon regeneration response. Recent studies have demonstrated that the JNK MAP kinase (MAPK) pathway, consisting of MLK-1 (MAPKKK)–MEK-1 (MAPKK)–KGB-1 (JNK MAPK), plays a crucial role in axon regeneration in *C*. *elegans* [4]. This JNK cascade can be inactivated at the MAPK activation step by members of the MAPK phosphatase (MKP) family [5]. The *C*. *elegans* MKP VHP-1 negatively regulates the MLK-1–MEK-1–KGB-1 JNK pathway [6]. *vhp-1* mutant animals are arrested during larval development due to hyperactivation of the JNK pathway. Indeed, the *vhp-1* larval arrest phenotype is suppressed by loss-of-function mutations of the *mlk-1*, *mek-1* or *kgb-1* gene [6]. In a previous effort to identify additional components involved in KGB-1 JNK-mediated signaling, we executed a genome-wide RNAi screen for suppressors of *vhp-1* lethality. From this RNAi screen, we isolated a number of *svh* (**s**uppressor of ***vh****p-1*) genes [7]. Among them, the *svh-1* and *svh-2* genes encode a growth factor and its cognate receptor tyrosine kinase (RTK), respectively. This SVH-1–SVH-2 signaling cascade positively regulates axon regeneration through tyrosine phosphorylation of MLK-1 in the KGB-1 pathway (Fig 1A) [7]. The *C*. *elegans* Shc adaptor protein SHC-1 is an essential component of the KGB-1 pathway that acts as an adaptor to link MEK-1 to MLK-1. The interaction between SHC-1 and MLK-1 depends on SVH-2-mediated phosphorylation of the Tyr residue in MLK-1 [8].

The ability of neurons to regenerate their axons following injury is modulated by interactions between the intrinsic axon growth machinery and the local extracellular environment. In the present study, we investigate the role of the *svh-4*/*ddr-2* gene in the regulation of axon regeneration. The *ddr-2* gene encodes an RTK that contains a discoidin domain activated by collagen. We demonstrate a link between the extracellular matrix and cell intrinsic pathways in the regulation of axonal regeneration.

## Results

### DDR-2 RTK containing a discoidin domain is required for efficient axon regeneration

The *svh-4* gene was identified as the *ddr-2* gene, which encodes a protein homolog of the discoidin domain receptors (DDRs) (Fig 1B and S1 Fig). DDRs are a subfamily of transmembrane collagen-binding RTKs [9]. DDRs are distinguished from other RTKs by a discoidin domain in their extracellular region [9]. There are two genes that encode DDRs in *C*. *elegans*, DDR-1 and DDR-2 (Fig 1B) [10]. They display a similar topology to the human homologs, with an extracellular region containing the ligand-binding discoidin domain, a single transmembrane region, and a cytoplasmic region which contains a tyrosine kinase domain.

In order to determine whether DDR functions in axon regeneration in vivo, we examined axon regeneration in *ddr-2* loss-of-function mutants. The *ok574* deletion allele disrupts the kinase domain and the *ddr-2(tm797)* knock-out causes a premature translation stop in the extracellular domain (Fig 1B) [10]. We assayed axon regeneration in the gamma-aminobutyric acid (GABA)-releasing D-type motor neurons of *C*. *elegans* using single-neuron laser axotomy (Fig 1C) [2]. In young adult wild-type animals, axons severed by laser were able to initiate regeneration within 24 hr (Fig 1C and 1D and S1 Table). At 24 hr after axon injury, the frequency of axon regeneration in *ddr-2(ok574)* and *ddr-2(tm797)* mutants was reduced (Fig 1C and 1D and S1 Table). In contrast, the *ddr-1(ok874)* deletion mutation (Fig 1B) had no effect on axon regeneration itself, but weakly enhanced the defective regeneration observed in *ddr-2(ok574)* mutants (Fig 1D and S1 Table). Expression of *ddr-1* cDNA under the control of the *unc-25* promoter failed to suppress the *ddr-2* defect (Fig 1D and S1 Table). Thus, DDR-2 plays a major role in the regulation of axon regeneration. The morphology of D-type motor neurons was normal in these two *ddr-2* mutants. To examine the effect of *ddr-2* on the regeneration dynamics, we monitored severed axons at a later time point (72 hr) after ablation. We observed that the frequency of axon regeneration in *ddr-2(ok574)* mutants was increased at 72 hr after axon injury, whereas regeneration in *svh-2(tm737)* mutants was not (Fig 1E and S1 Table). Thus, in *ddr-2* mutants, initiation of axon regeneration after laser surgery is delayed. Although *ddr-2* mutant animals exhibit axon guidance defects in major longitudinal tracts, most prominently in the ventral nerve cord [10], the *ddr-2* mutation had little effect on the regeneration guidance of vertical commissures of D-type motor neurons.

The *ddr-2* gene generates two transcripts from distinct start exons, which encode DDR-2a and DDR-2b, respectively [10]. However, since the DDR-2b form lacks the signal sequence in the N-terminal region, we chose to characterize the DDR-2a form. To confirm that the defect in axon regeneration was caused by the *ddr-2* mutation, we constructed a *Pddr-2a*::*ddr-2a* transgene, in which the *ddr-2a* cDNA was fused to its own promoter region of about 4.6 kb. Introduction of the *Pddr-2a*::*ddr-2a* transgene as an extrachromosomal array significantly rescued the defect associated with the *ddr-2(ok574)* mutation (Fig 1D and S1 Table). Therefore, in this study, we refer to *ddr-2a* as *ddr-2*. To test whether DDR-2 can act in a cell-autonomous manner, we expressed *ddr-2* cDNA driven by the *unc-25* promoter in *ddr-2* mutants. The *ddr-2* defect was rescued by expression of *ddr-2* in D-type motor neurons by the *unc-25* promoter (Fig 1D and S1 Table). These results demonstrate that DDR-2 functions cell-autonomously in D-type motor neurons. Consistent with this, we found that the *ddr-2* gene is expressed in D-type motor neurons using the transgene *Pddr-2*::*nls*::*venus*, which consists of the *ddr-2* promoter driving the fluorescent protein VENUS fused to the nuclear localization signal (NLS) (S2 Fig).

### The collagen EMB-9 is involved in DDR-2-mediated axon regeneration

DDRs are unique members of the family of RTKs in that they bind to and are activated by triple-helical collagen, the major component of the extracellular matrix (ECM). The Arg-105 residue in the discoidin domain of mammalian DDR2 is essential for collagen binding [11]. Sequence comparison suggested that the Arg-100 residue of *C*. *elegans* DDR-2 corresponds to Arg-105 of the mammalian DDR2 (S1A Fig) and therefore might also serve as a collagen binding site. To ask if the Arg-100 residue is important for DDR-2 function, we generated the *ddr-2* mutant [*ddr-2(R100A)*], in which Arg-100 is replaced with Ala. We found that the DDR-2(R100A) mutated form failed to rescue the defect in axon regeneration (Fig 1D and S1 Table). Thus, the Arg-100 collagen binding site is essential for the function of DDR-2 in axon regeneration following laser ablation.

Basement membrane collagens are likely to be candidates as ligands for DDR-2. In *C*. *elegans*, three collagens are associated with the basement membrane: the type IV collagens EMB-9 and LET-2 [12] and the type XV/XVIII collagen CLE-1 [13]. We found that at 24 hr after axon injury, the frequency of axon regeneration in *emb-9(g34*) temperature-sensitive embryonic lethal mutants [14] was reduced at the semi-permissive temperature of 20°C (Fig 1F and S1 Table). Similar to *ddr-2* mutants, we observed that the frequency of axon regeneration in *emb-9(g34*) mutants was increased at 72 hr after axon injury (Fig 1E and S1 Table). Furthermore, the regeneration defects seen in *emb-9(g34*); *ddr-2(ok574)* double mutants was no greater than those observed in the individual mutants (Fig 1F and S1 Table), suggesting that EMB-9 and DDR-2 act in the same genetic pathway. In addition, overexpression of *ddr-2* by the *unc-25* promoter suppressed the *emb-9* defect (Fig 1F and S1 Table), supporting the possibility that the EMB-9 collagen activates DDR-2 in axon regeneration. In contrast to *emb-9*, the *cle-1(cg120)* deletion mutation had no effect on axon regeneration (Fig 1F and S1 Table). DDR-1 and DDR-2 are required in the left ventral nerve cord pioneer neuron PVPR for the proper guidance [10]. The basement membrane component NID-1/Nidogen and the non-classical cadherin FMI-1/Flamingo also have been implicated in the navigation of PVPR [15–17]. The DDRs act in the same genetic pathway as NID-1 and FMI-1 with respect to PVPR axon guidance [10]. However, we found that neither *nid-1(cg119)* nor *fmi-1(rh308)* affected axon regeneration (Fig 1F and S1 Table).

### DDR-2 tyrosine kinase activity is essential for its function in axon regeneration

**Fig 2:**
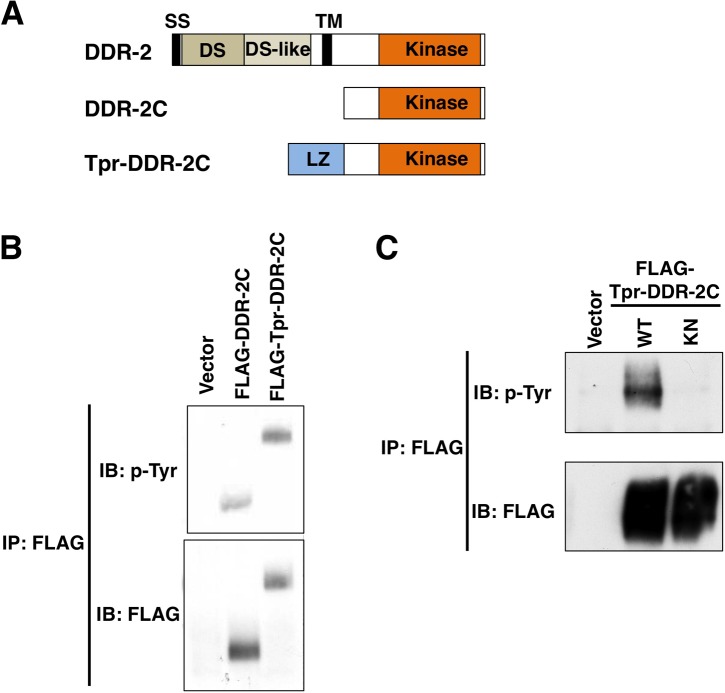
Tyrosine kinase activity of DDR-2. (A) Schematic diagrams of DDR-2, DDR-2C and Tpr-DDR-2C. LZ, leucine zipper. (B,C) Auto-tyrosine-phosphorylation of DDR-2. COS-7 cells were transfected with control vector or the plasmids encoding FLAG-DDR-2C, FLAG-Tpr-DDR-2C (WT) and FLAG-Tpr-DDR-2C (K554E) (KN), as indicated. Cell lysates were immunoprecipitated with anti-FLAG antibody (IP: FLAG) and immunoblotted with anti-phospho-tyrosine (pY) and anti-FLAG antibodies.

Upon binding to collagen, DDR is autophosphorylated by tyrosine kinase activity present in the receptor cytoplasmic tail. Therefore, measurement of DDR tyrosine phosphorylation is used to demonstrate its activation [18]. Next, we investigated whether DDR-2 possesses tyrosine kinase activity. When the cytoplasmic domain (amino acids 407–797) of DDR-2 (DDR-2C) (Fig 2A) was expressed in mammalian COS-7 cells and subjected to Western blotting analysis with anti-phospho-tyrosine (pY) antibody, we observed a small amount of tyrosine phosphorylation, suggesting tyrosine kinase activity (Fig 2B). We generated a constitutively active form of DDR-2C by fusing the N-terminus of a dimerization leucine zipper motif (Tpr; translocated promoter region) directly to DDR-2C (Fig 2A). The resulting hybrid protein is expected to form a dimer via the Tpr leucine zipper and to be constitutively activated in the absence of ligand [19,20]. When the FLAG-Tpr-DDR-2C hybrid protein was expressed in COS-7 cells, Tpr-DDR-2C was phosphorylated at tyrosine residues (Fig 2B and 2C). A catalytically inactive mutant [Tpr-DDR-2C(K554E)], in which Lys-554 was mutated to Glu (S1B Fig), exhibited no Tyr phosphorylation activity (Fig 2C), suggesting that Tpr-DDR-2C autophosphorylates at tyrosine residues. We next addressed the biological importance of DDR-2 kinase activity. When the catalytically inactive mutant *ddr-2(K554E)* was expressed under the control of the *unc-25* promoter in *ddr-2* mutants, the defect in axon regeneration was not rescued (Fig 1D and S1 Table). Thus, kinase activity is important for DDR-2 function in axon regeneration.

### Localization of DDR-2

**Fig 3:**
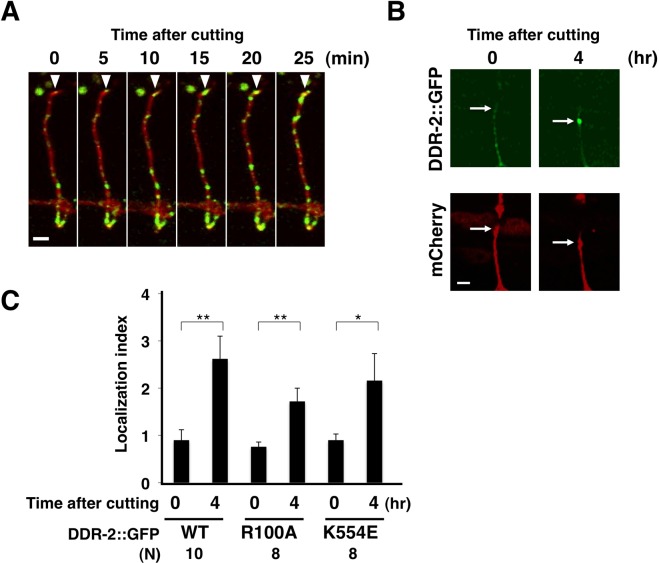
Localization of DDR-2. (A,B) Localization of DDR-2::GFP in D-type motor neurons after axon injury. Fluorescent images of severed axons in wild-type animals carrying *Punc-25*::*ddr-2*::*gfp* and *Punc-47*::*mcherry* are shown. Each image was taken at the indicated times after laser surgery. Arrowheads indicate the ends of proximal axons (A). The image was taken at 4 hr after axon injury. Arrows indicate the tip of the proximal injured axon (B). Scale bars = 2 μm. (C) Quantification of the relative fluorescence levels of DDR-2::GFP on the tips of the severed axons. The relative fluorescent intensities of severed axons in animals carrying *Punc-25*::*ddr-2*::*gfp* and *Punc-47*::*mcherry*::*caax* were compared. “N” = number of axons. Error bars indicate 95% CI. *P<0.05, **P<0.01.

We next examined DDR-2 localization in D-type motor neurons during axon regeneration. For this purpose, we expressed the *Punc-25*::*ddr-2*::*gfp* gene in wild-type animals and performed fluorescent imaging analysis in D neurons. We confirmed that DDR-2::GFP is functional, because the *Punc-25*::*ddr-2*::*gfp* gene was able to rescue the *ddr-2* mutant phenotype (Fig 1D and S1 Table). At 0 min after axon injury, we observed that DDR-2::GFP was distributed in a punctate pattern throughout D-type motor axons (Fig 3A). Following laser ablation of the axons, the amounts of DDR-2::GFP increased at different puncta locations along the axon as well as at the severed end (Fig 3A). At 4 hr after axon injury, significant amounts of DDR-2::GFP accumulated at the proximal ends of the injured axons (Fig 3B). We quantified the fluorescence intensity ratios of DDR-2::GFP to a marker expressed in D neurons at the severed end. Since DDR-2::GFP is expected to be membrane bound, mCherry carrying the CAAX box derived from the *C*. *elegans* RAS-2 C-terminal CAAX sequence (Cys-Leu-Ile-Ser) was expressed in D neurons by the *unc-47* promoter and used as a membrane-bound marker. We confirmed enhanced localization of DDR-2::GFP at the severed end (Fig 3C). DDR-2(R100A)::GFP, the mutant defective in binding collagen, and a kinase-negative DDR-2(K554E)::GFP were localized at the proximal ends of the injured axons similar to the wild-type DDR-2 protein (Fig 3C). This indicates that, although the collagen-binding function and kinase activity are required for DDR-2 function in regeneration (Fig 1D), they are not required for the localization of DDR-2 at the severed end.

### DDR-2 functions upstream of SVH-1 and SVH-2 in the JNK pathway

**Fig 4:**
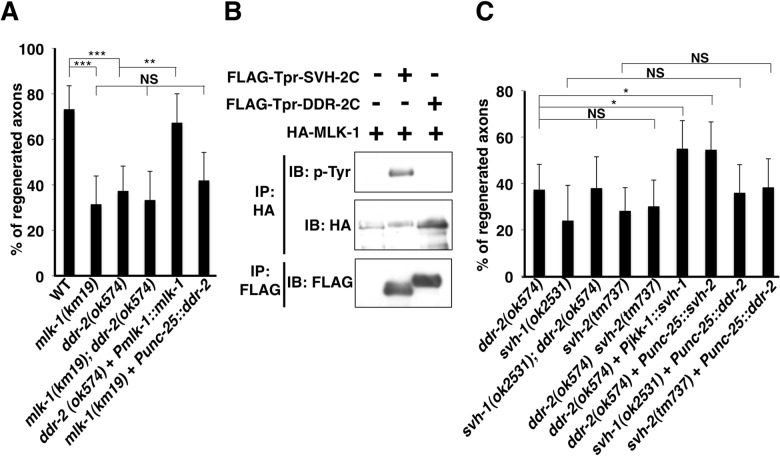
The relationship between DDR-2 and SVH-2 in axon regeneration. (A,C) Percentages of axons that initiated regeneration 24 hr after laser surgery. Error bars indicate 95% CI. *P<0.05, **P<0.01, ***P<0.001. NS, not significant. (B) Tyrosine phosphorylation of MLK-1. COS-7 cells were transfected with plasmids encoding FLAG-Tpr-SVH-2C, FLAG-Tpr-DDR-2C and HA-MLK-1, as indicated. Complexes immunoprecipitated with anti-HA or anti-FLAG antibody were analyzed by immunoblotting with anti-phospho-tyrosine (pY), anti-HA and anti-FLAG antibodies.

Since our RNAi screen for *svh* genes was originally designed to identify components functioning in the JNK pathway [7], we next investigated where DDR-2 acts in this pathway during axon regeneration. The *mlk-1* gene encodes a MAPKKK that functions in the JNK pathway (Fig 1A), and *mlk-1(km19)* null mutants are defective in axon regeneration [4]. We found that the *ddr-2(ok574)* mutation did not enhance the regeneration defect in *mlk-1(km19)* mutants (Fig 4A and S1 Table), suggesting that DDR-2 and MLK-1 act in the same pathway. We next examined the epistatic relationship between DDR-2 and MLK-1. Overexpression of wild-type *mlk-1* by its own promoter suppressed the regeneration defect in *ddr-2(ok574)* mutants, whereas overexpression of *ddr-2* by the *unc-25* promoter failed to suppress the *mlk-1* defect (Fig 4A and S1 Table). These results suggest that DDR-2 functions upstream of MLK-1 in axon regeneration.

We have previously demonstrated that the SVH-2 RTK receptor, which is activated by the SVH-1 growth factor, mediates activation of the MLK-1–MEK-1–KGB-1 JNK cascade by phosphorylating MLK-1 following axonal injury (Fig 1A) [7]. The above genetic interaction between DDR-2 and MLK-1 raised the possibility that DDR-2 might also phosphorylate MLK-1. To test this possibility, we co-expressed FLAG-Tpr-DDR-2C and HA-MLK-1 in COS-7 cells. We found that in contrast to Tpr-SVH-2C, Tpr-DDR-2C was unable to phosphorylate MLK-1 (Fig 4B). These results suggest that, unlike SVH-2, DDR-2 does not act on MLK-1.

We next examined the relationship of DDR-2 with the SVH-1–SVH-2 pathway. SVH-1 is constitutively expressed in ADL sensory neurons and expression of SVH-2 is induced by axon injury [7]. Animals harboring *ddr-2*; *svh-1* or *ddr-2 svh-2* double mutations had a regeneration frequency indistinguishable from that observed in the *ddr-2* single mutants (Fig 4C and S1 Table). Thus, DDR-2 acts in the same pathway with SVH-1 and SVH-2. Furthermore, overexpression of *svh-1* under the control of a pan-neuronal promoter *Pjkk-1* or that of *svh-2* by the *unc-25* promoter suppressed the regeneration defect in *ddr-2* mutants, whereas overexpression of *ddr-2* by the *unc-25* promoter failed to suppress the *svh-1* or *svh-2* defect (Fig 4C and S1 Table). These results suggest that DDR-2 functions upstream of SVH-1 and SVH-2.

### The scaffold protein SHC-1 functions between DDR-2 and SVH-2 in axon regeneration

**Fig 5:**
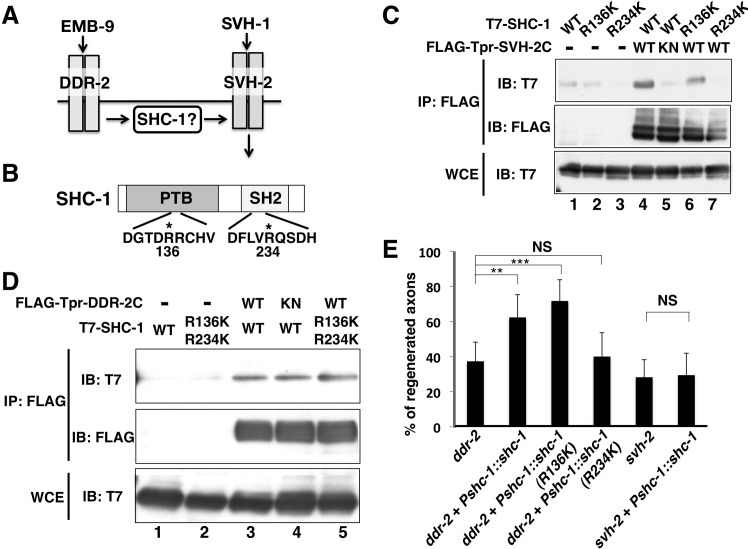
Interactions of SHC-1 with SVH-2 and DDR-2. (A) The relationship among EMB-9–DDR-2, SHC-1 and SVH-1–SVH-2 in the JNK signaling pathway. (B) Structure of SHC-1. Dark and hatched boxes represent the PTB and SH2 domains, respectively. Essential Arg residues required for binding to phospho-tyrosine in PTB and SH2 domains are indicated by asterisks. (C,D) Interactions of SHC-1 with SVH-2 and DDR-2. COS-7 cells were transfected with plasmids encoding FLAG-Tpr-SVH-2C (WT), FLAG-Tpr-SVH-2C(K767R) (KN), FLAG-Tpr-DDR-2C (WT), FLAG-Tpr-DDR-2C (K554E) (KN), T7-SHC-1 (WT), T7-SHC-1 (R136K), T7-SHC-1 (R234K) and T7-SHC-1 (R136K; R234K), as indicated. Whole-cell extracts and immunoprecipitated complexes obtained with anti-FLAG antibody (IP: FLAG) were analyzed by immunoblotting. (E) Percentages of axons that initiated regeneration 24 hr after laser surgery. Error bars indicate 95% CI. **P<0.01, ***P<0.001. NS, not significant.

How does EMB-9 collagen–DDR-2 RTK act upstream of the SVH-1 growth factor–SVH-2 RTK pathway (Fig 5A)? We first examined the role of the *C*. *elegans* Shc adaptor protein SHC-1, which is a crucial component of the KGB-1 pathway and required for axon regeneration [8]. The SHC-1 protein contains a phosphotyrosine binding (PTB) domain and a Src homology 2 (SH2) domain in the N-terminal and C-terminal regions, respectively (Fig 5B) [21]. Therefore, we examined the possibility that Tyr-autophosphorylation of SVH-2 or DDR-2 creates a binding site for SHC-1. When T7-SHC-1 was co-expressed with FLAG-Tpr-SVH-2C in mammalian COS-7 cells, immunoprecipitation analysis revealed that SVH-2 associated with SHC-1 (Fig 5C, lane 4). However, a catalytically inactive mutant form of SVH-2, Tpr-SVH-2C(K767R), was unable to associate with SHC-1 (Fig 5C, lane 5), suggesting that the interaction between SVH-2 and SHC-1 is dependent on the Tyr-autophosphorylation of SVH-2. To test whether the PTB and/or SH2 domains in SHC-1 are essential for its interaction with SVH-2, we generated mutations in each. To inactivate the PTB domain, Arg-136 was mutated to Lys (R136K), and to inactivate the SH2 domain, Arg-234 was mutated to Lys (R234K) (Fig 5B). We found that Tpr-SVH-2C associated with SHC-1(R136K) but not with SHC-1(R234K) (Fig 5C, lanes 6 and 7), indicating that the C-terminal SH2 domain of SHC-1 is important for its interaction with SVH-2.

We also found that the cytoplasmic domain, DDR-2C, was able to interact with SHC-1 (S3 Fig), even though DDR-2C exhibits only weak kinase activity (Fig 2B). These results suggest that the interaction between DDR-2C and SHC-1 does not depend on DDR-2C Tyr-autophosphorylation. Consistent with this, Tpr-DDR-2C interacted with SHC-1 in a manner independent of its tyrosine kinase activity (Fig 5D, lanes 3 and 4). Furthermore, Tpr-DDR-2C still associated with the SHC-1(R136K; R234K) mutant, in which both residues were mutated (Fig 5D, lane 5). Thus, the association with SHC-1 is different between DDR-2 and SVH-2.

The above results raised the possibility that SHC-1 functions between DDR-2 and SVH-2 in axon regeneration. To test this, we used a genetic bypass assay to examine the position of *shc-1* relative to *ddr-2* and *svh-2* in the axon regeneration pathway. First, we asked whether overexpression of *shc-1* could bypass the functional requirement for *ddr-2* or *svh-2* in axon regeneration. We found that overexpression of *shc-1* by its own promoter rescued the axon regeneration defect of *ddr-2* mutants, but not of *svh-2* mutants (Fig 5E and S1 Table). We next investigated the functional significance of the SHC-1–SVH-2 interaction in the rescue of the *ddr-2* defect by *shc-1* overexpression. When *shc-1(R234K)*, a mutant defective in its interaction with SVH-2, was overexpressed, it failed to suppress the axon regeneration defect observed in *ddr-2* mutants (Fig 5E and S1 Table). In contrast, when SHC-1(R136K), which has the ability to associate with SVH-2, was overexpressed from its own promoter, it was able to suppress the *ddr-2* defect (Fig 5E and S1 Table). These results suggest that the binding of SHC-1 with SVH-2 is important for SVH-2 activity in the axon regeneration pathway.

## Discussion

Communication between cells and their environment is mediated by specific cell surface receptors that transduce signals from the outside of the cell to the inside. One class of receptors that plays a particularly crucial role in many fundamental cellular processes is the RTKs [22]. We have investigated one subgroup of RTKs, the DDRs, which are characterized by the presence of a discoidin domain. The DDRs have been shown to bind to, and be activated by, basement membrane collagens [18], raising the possibility that basement membrane collagens are the normal ligands for DDRs. Mouse DDR1 functions in neurite elongation in granule neurons, which is likely induced by the interaction between DDR1 on granule cells and collagen on the pial layer of the developing cerebellum [23]. In contrast to DDR1 function in the cerebellum of mammals, Unsoeld *et al*. demonstrated that *C*. *elegans ddr-1* and *ddr-2* loss-of-function mutations have no effect on axonal extension [10]. However, they did observe that *ddr-2* mutant animals exhibit axon guidance defects in major longitudinal tracts, most prominently in the ventral nerve cord [10]. Mammalian DDR1 is also required for arterial wound repair after injury [24]. In this study we examined the role of DDR-2 in the regeneration of injured axons in *C*. *elegans*. We found that the *ddr-2* mutation delayed initiation of regeneration in D-type motor neurons after laser surgery. Similarly, the *emb-9* mutation defective in the type IV collagen affected the initiation of axon regeneration. Furthermore, we demonstrated that overexpression of *ddr-2* was able to suppress the *emb-9* defect. These genetic interaction studies suggest that EMB-9 acts as a ligand for DDR-2 in this process, and indicate that basement membrane collagen can affect axon regeneration. Thus, our study identified a novel role for DDR in axon regeneration in the *C*. *elegans* nervous system.

**Fig 6:**
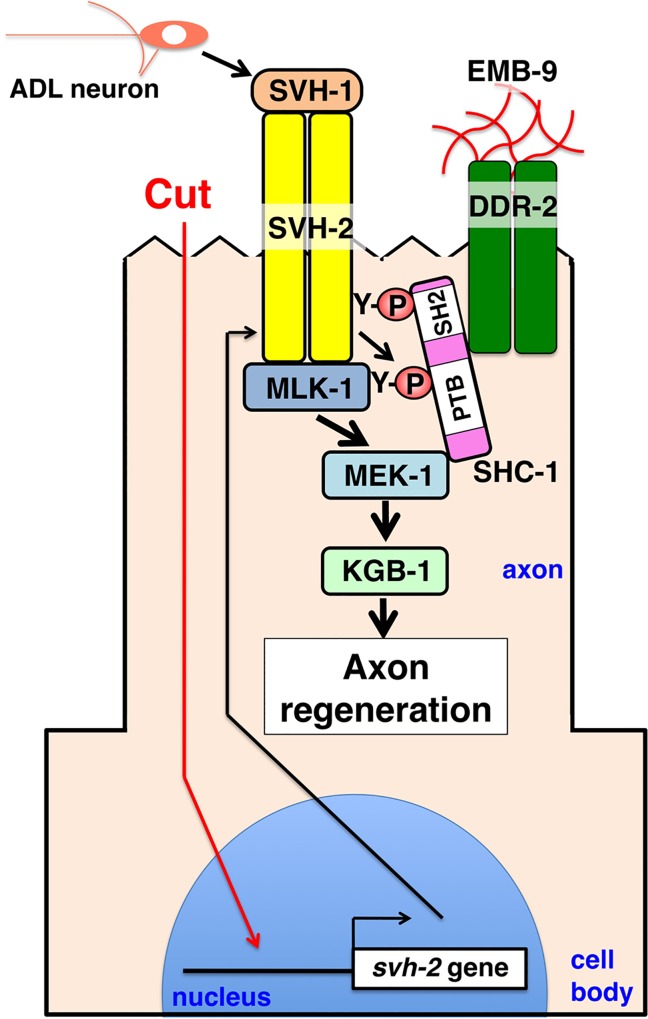
DDR-2 modulates SVH-1–SVH-2 signaling to regulate axon regeneration after neuron injury. HGF/plasminogen-like protein SVH-1 is constitutively expressed in, and secreted from ADL sensory neurons in the head. Expression of SVH-2 is induced by axon injury. Following axon injury, DDR-2 accumulates at the severed end and is activated by EMB-9. DDR-2 facilitates the efficient activation of SVH-2 by SVH-1. Activated SVH-2 phosphorylates MLK-1 MAPKKK at a tyrosine residue, creating a docking site for SHC-1. SHC-1 constitutively forms a complex with MEK-1 MAPKK, and thereby functions to connect MLK-1 and MEK-1. SHC-1 participates in restricting the SVH-1–SVH-2 signal to the MLK-1–MEK-1 pathway.

The observation that DDRs are activated by an ECM protein, triple-helical collagen, raises the question of how cell surface receptors for ECM components affects the axon regeneration pathway. The activation of DDR kinases by collagens follows a delayed time course relative to conventional growth factor receptors, consistent with the possibility that DDRs monitor the relationship of the cell to the ECM over the long term, rather than mediating an acute signaling response. One model envisions DDR-2 acting on the surface of regenerating axons as a sensor for the ECM via its recognition of collagen. Our genetic data suggest that DDR-2, the *C*. *elegans* Shc adaptor protein SHC-1, and the HGF-like RTK SVH-2 function in the same genetic pathway regulating axon regeneration. When DDR-2 binds to collagen, SHC-1 acts as a bridging molecule linking SVH-2 to the DDR-2 cytoplasmic domain. This triggers SVH-1 growth factor-mediated activation of SVH-2, which in turn transmits the initial injury signal, leading to the activation of JNK (Fig 6). SVH-1 and SVH-2 are essential for axon regeneration, whereas EMB-9 and DDR-2 are required for the efficient initiation of axon regeneration but are not essential for it. Thus, EMB-9–DDR-2 modulate the SVH-2–JNK pathway in response to axon injury.

The SHC-1 protein contains PTB and SH2 domains [21]. These domains control many cellular activities by binding to tyrosine-phosphorylated peptides [25]. Tyrosine phosphorylation signaling often originates from the cell surface through activation of RTKs. In mammals, RTKs generate diverse patterns of phosphorylation on their cytosolic tails, as well as on other associated proteins. These phosphorylation patterns are recognized mainly by proteins containing a PTB or SH2 domain, which are recruited to phospho-tyrosine sites [25]. The *C*. *elegans* scaffold protein SHC-1 binds via its SH2 domain to Tyr residues autophosphorylated in SVH-2-RTK. Additionally, SVH-2 trans-phosphorylates MLK-1 MAPKKK at a tyrosine residue, creating a docking site for the PTB domain of SHC-1 [8]. SHC-1 constitutively forms a complex with MEK-1 MAPKK, and thereby functions to connect MLK-1 and MEK-1 (Fig 6). Thus, SHC-1 could potentially interact with multiple phospho-tyrosine sites that are proximal to one another. These data suggest that SHC-1 participates in restricting the SVH-1–SVH-2 signal to the MLK-1–MEK-1 pathway. In summary, our findings describe a novel mechanism involving DDR-2 modulation of the SVH-1–SVH-2 signaling pathway to regulate axon regeneration after neuronal injury. The key component of this pathway is the SHC-1 adaptor protein, which selectively links the DDR-2 RTK with the activation of SVH-2 RTK signaling. This study is the first to report the cooperation between two different types of RTKs, DDR-2 and SVH-2, to promote axon regeneration.

## Materials and Methods

### *C*. *elegans* strains

The *C*. *elegans* strains used in this study are listed in S2 Table. All strains were maintained on nematode growth medium (NGM) plates and fed with bacteria of the OP50 strain, as described previously [26].

### Plasmids

The *Pddr-2a*::*nls*::*venus* plasmid was made by amplifying approximately 4.6 kb of the *ddr-2a* promoter from the N2 genome by PCR and inserting it into the pPDnlsVenus vector [7]. *Punc-25*::*ddr-2a* was generated by inserting the *ddr-2a* cDNA derived from yk clones (gifts from Dr. Y. Kohara) into the pSC325 vector. *Pddr-2a*::*ddr-2a* was made by replacing the *nls*::*venus* fragment of *Pddr-2a*::*nls*::*venus* with *ddr-2a* cDNA. *Punc-25*::*ddr-2a(R100A)* and *Punc-25*::*ddr-2a(K554E)* were generated by oligonucleotide-directed PCR using *Punc-25*::*ddr-2a* as a template and verified by DNA sequencing. To make the *Punc-25*::*ddr-2a*::*gfp* plasmid, a modified *ddr-2a* cDNA that deletes the termination codon was generated by PCR and inserted into the pSC325 vector with a *gfp* gene from pPD95.75. *Punc-25*::*ddr-2a(R100A)*::*gfp* and *Punc-25*::*ddr-2a(K554E)*::*gfp* plasmids were made by replacing a portion of the *ddr-2a* cDNA in the *Punc-25*::*ddr-2a*::*gfp* plasmid with the corresponding fragment of *ddr-2a(R100A)* cDNA and *ddr-2a(K554E)* cDNA, respectively. *Punc-25*::*ddr-1* was generated by inserting the *ddr-1* cDNA, which was amplified from a *C*. *elegans* cDNA library [27]. To make the FLAG-DDR-2C plasmid, a 1.2 kb cDNA fragment encoding amino acids 407–797 of the DDR-2 protein (DDR-2C) was inserted into the pCMV-FLAG vector. To make the FLAG-Tpr-DDR-2C plasmid, a 1.2 kb cDNA fragment encoding DDR-2C was inserted together with a 0.4 kb cDNA fragment encoding amino acids 1–142 of human Tpr protein [7] into the pCMV-FLAG vector. The FLAG-Tpr-DDR-2(K554E) plasmid was constructed by oligonucleotide-directed PCR using FLAG-Tpr-DDR-2 as a template and verified by DNA sequencing. *Punc-25*::*nes*::*cfp* was constructed by inserting 300 bp of the *jkk-1* cDNA [27], which encodes the N-terminal 100 amino acids of JKK-1 carrying two nuclear export signals, into the *Punc-25*::*cfp* plasmid. *Punc-47*::*mcherry*::*caax* plasmid was generated by inserting the C-terminal 12 amino acids of *C*. *elegans ras-2* gene (KKRKDKGKCLIS) into the region just prior to the termination codon of mCherry ORF in *Punc-47*::*mcherry* by PCR. *Pshc-1*::*shc-1*, *Pshc-1*::*shc-1(R136K)*, *Pshc-1*::*shc-1(R234K)*, *Pmyo-2*::*dsredmonomer*, FLAG-Tpr-SVH-2C, FLAG-Tpr-SVH-2C(K767R), HA-MLK-1, T7-SHC-1 and T7-SHC-1(R136K; R234K) plasmids were described previously [21].

### Transgenic animals

Transgenic animals were obtained by the standard *C*. *elegans* microinjection method [28]. *Pmyo-2*::*dsredmonomer*, *Punc-47*::*mcherry*, *Punc-47*::*mcherry*::*caax*, *Punc-25*::*nes*::*cfp*, *Pddr-2a*::*ddr-2a*, *Punc-25*::*ddr-2a*, *Punc-25*::*ddr-2a(R100A)*, *Punc-25*::*ddr-2a(K554E)*, *Punc-25*::*ddr-1*, *Punc-25*::*svh-2*, *Pshc-1*::*shc-1*, *Pshc-1*::*shc-1(R234K)*, *Pshc-1*::*shc-1(R136K)*, *Punc-25*::*ddr-2a*::*gfp*, *Pddr-2a*::*nls*::*venus*, *Punc-25*::*ddr-2a(R100A)*::*gfp* and *Punc-25*::*ddr-2a(K554E)*::*gfp* plasmids were used in *kmEx1181* [*Pshc-1*::*shc-1* (50 ng) *+ Pmyo-2*::*dsredmonomer* (25 ng)], *kmEx1201* [*Pddr-2a*::*ddr-2a* (50 ng) + *Pmyo-2*::*dsredmonomer* (25 ng)], *kmEx1202* [*Punc-25*::*ddr-2a* (25 ng) + *Pmyo-2*::*dsredmonomer* (25 ng)], *kmEx1204* [*Punc-25*::*ddr-2a(R100A)* (25 ng) + *Pmyo-2*::*dsredmonomer* (25 ng)], *kmEx1205* [*Punc-25*::*ddr-2a(K554E)* (25 ng) + *Pmyo-2*::*dsredmonomer* (25 ng)], *kmEx1206* [*Punc-25*::*svh-2* (50 ng) + *Pmyo-2*::*dsredmonomer* (25 ng)], *kmEx1207* [*Pshc-1*::*shc-1(R234K)* (50 ng) + *Pmyo-2*::*dsredmonomer* (25 ng)], *kmEx1208* [*Pshc-1*::*shc-1(R136K)* (50 ng) + *Pmyo-2*::*dsredmonomer* (25 ng)], *kmEx1209* [*Punc-25*::*ddr-2a*::*gfp* (25 ng) + *Punc-47*::*mcherry* (50 ng) + *Pmyo-2*::*dsredmonomer* (25 ng)], *kmEx1210* [*Pddr-2a*::*nls*::*venus* (25 ng) + *Punc-25*::*nes*::*cfp* (50 ng) + *Pmyo-2*::*dsredmonomer* (25 ng)], *kmEx1213* [*Punc-25*::*ddr-1* (25 ng) + *Pmyo-2*::*dsredmonomer* (25 ng)], *kmEx1214* [*Punc-25*::*ddr-2a*::*gfp* (25 ng) + *Punc-47*::*mcherry*::*caax* (50 ng) + *Pmyo-2*::*dsredmonomer* (25 ng)], *kmEx1215* [*Punc-25*::*ddr-2a(R100A)*::*gfp* (25 ng) + *Punc-47*::*mcherry*::*caax* (50 ng) + *Pmyo-2*::*dsredmonomer* (25 ng)], and *kmEx1216* [*Punc-25*::*ddr-2a(K554E)*::*gfp* (25 ng) + *Punc-47*::*mcherry*::*caax* (50 ng) + *Pmyo-2*::*dsredmonomer* (25 ng)]. The *kmEx505* and *kmEx507* extrachromosomal arrays have been previously described [7].

### Axotomy

Axotomy was performed as previously described [7]. Young adult hermaphrodite animals were immobilized with 0.7% sodium azide or 20 mM levamisole solution in M9 buffer on a 2% agarose pad under a cover slip. D-type motor neurons expressing GFP were imaged with a fluorescence microscope. Selected D-type neurons were severed using a 440-nm MicroPoint ablation Laser System from Photonic Instruments. The animals were transferred to an agar plate and remounted for fluorescent imaging ~24 hr after surgery. Proximal axon segments that showed no change after 24 hr were counted as “no regeneration”. Axons that grew a distance of 5 μm or more were scored as “regenerated”. Indeed, neurons that regrew below 5 μm did not initiate regrowth at all. At least 20 animals with 1–2 axotomized commissures were observed for most experiments.

### Microscopy

Standard fluorescent images of transgenic animals were observed under a X100 objective of a Nikon ECRIPSE E800 fluorescent microscope and photographed with a Hamamatsu 3CCD camera. Confocal fluorescent images were taken on an Olympus FV-500 confocal laser-scanning microscope with a X100 objective.

### Quantification of DDR-2::GFP

Localization of DDR-2::GFP fluorescence was quantified using the ImageJ program (NIH). First, regions to be analyzed were outlined with closed polygons and the fluorescent intensities of GFP and mCherry::CAAX expressed in D neurons by the *unc-47* promoter (*Punc-47*::*mcherry*::*caax*) within these areas were determined. Then each net fluorescent intensity was calculated by subtracting the background fluorescence outside of the neurons. The relative fluorescent intensity (RFI) for each region was obtained by dividing the value of GFP fluorescence with that of mCherry fluorescence in the same region. Then the RFI at the proximal end of the injured axon was divided by the RFI at the axon region below the proximal end in the same axon.

### Statistical analysis

Statistical analyses were carried out as described previously [8]. Briefly, confidence intervals (95%) were calculated by the modified Wald method and two-tailed P values were calculated using Fisher’s exact test (http://www.graphpad.com/quickcalcs/). The Welch’s *t* -test was performed using a *t*-test calculator (http://www.graphpad.com/quickcalcs/ttest1/).

### Immunoblotting

Transfection of transgenes into COS-7 cells, preparation of the cell lysates, immunoprecipitation and immunoblotting using anti-FLAG, ant-HA, anti-T7 and anti-phospho-tyrosine antibodies have been described previously [7].

## Supporting Information

S1 Fig (A) Discoidin (DS) and DS-like domains. The DS domain is boxed. Identical and similar residues are highlighted with black and gray shading, respectively. Conserved Arg residue targeted for mutagenesis in this study is indicated by asterisk. (B) Protein kinase domain. Conserved Lys residue targeted for mutagenesis in this study is indicated by asterisk.
(PDF)

S2 Fig Fluorescent images of animals carrying the *Pddr-2*::*nls*::*venus* and *Punc-25*::*nes*::*cfp* transgenes at L3 stage are shown. Yellow arrowheads indicate the positions of nuclei. NLS::VENUS and NES::CFP are localized to the nucleus and cytoplasm, respectively, in D neurons. Scale bar = 10 μm.
(PDF)

S3 Fig COS-7 cells were transfected with plasmids encoding FLAG-DDR-2C and T7-SHC-1, as indicated. Whole-cell extracts and immunoprecipitated complexes obtained with anti-FLAG antibody (IP: FLAG) were analyzed by immunoblotting. An arrow indicates the position of FLAG-DDR-2C.
(PDF)

S1 Table Raw data for genotypes tested by axotomy. (PDF)

S2 Table Strains used in this study. (PDF)
