# Supplementary material for: The C. elegans Discoidin Domain Receptor DDR-2 Modulates the Met-like RTK–JNK Signaling Pathway in Axon Regeneration
Source: PLoS Genet. 2016 Dec 16;12(12):e1006475. doi: 10.1371/journal.pgen.1006475 (PMC5161311; doi:10.1371/journal.pgen.1006475)
Supplement: S1 Fig — (A) Discoidin (DS) and DS-like domains. The DS domain is boxed. Identical and similar residues are highlighted with black and gray shading, respectively. Conserved Arg residue targeted for mutagenesis in this study is indicated by asterisk. (B) Protein kinase domain. Conserved Lys residue targeted for mutagenesis in this study is indicated by asterisk. (PDF) [file pgen.1006475.s001.pdf]

A

```
                                                                                              *
C. elegans DDR-2  24 ECSHQLGMSNRKIRDEQISASSSFDLQSTGPQHARAHQESGSGAWCPKNQINSLS-KEWLQISFSVDTVITSVETQGRFD
Human DDR2        29 ICRYPLGMSGGQIPDEDITASSQWS-ESTAAKYGRLDSEEGDGAWCPEIPVEPDDLKEFLQIDLHTLHFITLVGTQGRHA

C. elegans DDR-2 103 DGRGMEYATAFKIQYWRPSLNAWASYKDDFELETIPANNDTEHAIRRHLDRAIIARRIRIVPVSNSTRTVCMRVEVFGCP
Human DDR2       108 GGHGIEFAPMYKINYSRDG-TRWISWRNRHGKQVLDGNSNPYDIFLKDLEPPIVARFVRFIPVTDHSMNVCMRVELYGCV

C. elegans DDR-2 188 FDDSLVFYN---VDQGDLQSG-ISY-HDFSYDGNLANSPHLTGGIGKLYDGEVGKNNVFVNH--HKW-----VGWRRKR-
Human DDR2       187 WLDGLVSYNAPAGQQFVLPGGSIIYLNDSVYDGAVGYS--MTEGLGQLTDGVSGLDDFTQTHEYHVWPGYDYVGWRNESA

C. elegans DDR-2 255 -NGNVKLAFEFSELRNISGILIHTSNEFKKSAKAFSSATVLFSINGKDFSDTIVHFNNPEDTESEVPRWIRIPVNNRIAK
Human DDR2       265 TNGYIEIMFEFDRIRNFTTMKVHCNNMFAKGVKIFKEVQCYFRSEASEWEPNAISFPLVLDDVNPSARFVTVPLHHRMAS

C. elegans DDR-2 334 VAKIRLNFGTDSDWLFISEVNFESNH
Human DDR2       345 AIKCQYHF-ADT-WMMFSEITFQSDA
```

B

```
                                                                *
C. elegans DDR-2 532 LICVSRIGQGEFGEVDLCQLENRK------------------VAVKKLHGISQADEFS-FHREIRVLGSLKHPNVV
Human DDR2       567 LTFKEKLGEGQFGEVHLCEVEGMEKFKDKDFALDVSANQPVLVAVKMLRADANKNARNDFLKEIKIMSRLKDPNII

C. elegans DDR-2 589 EVVGVCTIQKPILC-IMEYMENGDLKSYILK-NPTIQTSQCI-SIC-TQL-------AAGLAYLESCNFVHRDIAA
Human DDR2       643 HLLAVCITDDP-LCMITEYMENGDLNQFLSRHEPPNSSSDVRTVSYTNLKFMATQIASGMKYLSSLNFVHRDLAT

C. elegans DDR-2 654 RNCLVDGEGNVKIADFGMARSLYSQEYYKVEGKFVLPIRWMAWEALLGKFSTASDVWGFGVTMWEIFSLCSEKPY
Human DDR2       718 RNCLVGKNYTIKIADFGMSRNLYSGDYYRIQGRAVLPIRWMSWESILLGKFTTASDVWAFGVTLWETFTFCQEQPY

C. elegans DDR-2 730 SDMTDDDVVENLQSMSSTGSLKQVLSRPRMCPSKLYNEQILPCWNYESSRRPSFENVHLHL
Human DDR2       794 SQLSDEQVIENTGEFFRDQGRQTYLPQPAICPDSVYK-LMLSCWRRDTKNRPSFQEIHLLL
```
