# Supplementary figures and images for: The C. elegans Discoidin Domain Receptor DDR-2 Modulates the Met-like RTK–JNK Signaling Pathway in Axon Regeneration
Source: PLoS Genet. 2016 Dec 16;12(12):e1006475. doi: 10.1371/journal.pgen.1006475 (PMC5161311; doi:10.1371/journal.pgen.1006475)

*Punc-25::nes::cfp*

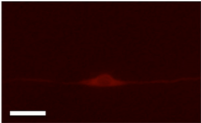

*Pddr-2::nls::venus*

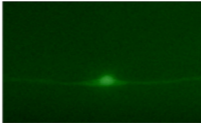

Merge

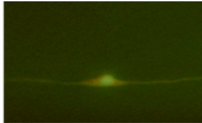

Supplement: S2 Fig — Yellow arrowheads indicate the positions of nuclei. NLS::VENUS and NES::CFP are localized to the nucleus and cytoplasm, respectively, in D neurons. Scale bar = 10 μm. (PDF) [file pgen.1006475.s002.pdf]

FLAG-DDR-2C

-

+

T7-SHC-1

+

+

IP: FLAG

IB: T7

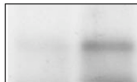

IB: FLAG

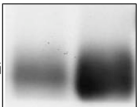

← FLAG-DDR-2C

WCE

IB: T7

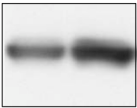

Supplement: S3 Fig — Whole-cell extracts and immunoprecipitated complexes obtained with anti-FLAG antibody (IP: FLAG) were analyzed by immunoblotting. An arrow indicates the position of FLAG-DDR-2C. (PDF) [file pgen.1006475.s003.pdf]
