# Supplementary material for: The C. elegans Discoidin Domain Receptor DDR-2 Modulates the Met-like RTK–JNK Signaling Pathway in Axon Regeneration
Source: PLoS Genet. 2016 Dec 16;12(12):e1006475. doi: 10.1371/journal.pgen.1006475 (PMC5161311; doi:10.1371/journal.pgen.1006475)
Supplement: S1 Table — (PDF) [file pgen.1006475.s004.pdf]

S1 Table

Strain	Genotype *(juIs76* background)	Regeneration	Total	% of regeneration	*P* value
KU501	wild type	52	71	73	-
KU1224	*ddr-1(ok874)*	31	55	56	0.059[a]
KU1201	*ddr-2(ok574)*	28	75	37	<0.001[a]
KU1228	*ddr-1(ok874) ddr-2(ok574)*	14	67	21	0.042[b]
KU1227	*ddr-2(ok574); Ex[Punc-25::ddr-1]*	21	54	39	0.856[b]
KU1202	*ddr-2(ok574); Ex[Pddr-2::ddr-2]*	36	61	59	0.016[b]
KU1203	*ddr-2(ok574); Ex[Punc-25::ddr-2]*	30	53	57	0.047[b]
KU1205	*ddr-2(ok574); Ex[Punc-25::ddr-2(R100A)]*	19	51	37	1[b]
KU1209	*ddr-2(ok574); Ex[Punc25::ddr-2(K554E)]*	21	52	40	0.853[b]
KU1206	*emb-9(g34)*	26	62	42	<0.001[a]
KU1207	*emb-9(g34); ddr-2(ok574)*	19	52	37	0.571[c]
KU1208	*emb-9(g34); Ex[Punc-25::ddr-2]*	33	54	61	0.043[c]
KU1233	*cle-1(cg120)*	39	61	64	0.264[a]
KU1234	*fmi-1(rh308)*	38	52	73	1[a]
KU1235	*nid-1(cg119)*	42	59	71	0.845[a]
KU1229	*ddr-2(ok574); Ex[Punc-25::ddr-2::gfp]*	40	58	69	<0.001[b]
KU504	*mlk-1(km19)*	17	54	31	<0.001[a]
KU1210	*mlk-1(km19); ddr-2(ok574)*	18	54	33	1[d]
KU1211	*ddr-2(ok574); Ex[Pmlk-1::mlk-1]*	35	52	67	0.001[b]
KU1212	*mlk-1(km19); Ex[Punc-25::ddr-2]*	26	62	42	0.256[d]
KU502	*svh-1(ok2531)*	10[k]	41[k]	24[k]	-
KU1232	*svh-1(ok2531); ddr-2(ok574)*	19	50	38	1[b]
KU503	*svh-2(tm737)*	22	78	28	<0.001[a]
KU1213	*ddr-2(ok574) svh-2(tm737)*	19	63	30	0.471[b]
KU1231	*ddr-2(ok574); Ex[Pjkk-1::svh-1]*	36	65	55	0.041[b]
KU1214	*ddr-2(ok574); Ex[Punc-25::svh-2]*	36	66	55	0.044[b]
KU1230	*svh-1(ok2531); Ex[Punc-25::ddr-2]*	22	61	36	0.278[f]
KU1215	*svh-2(tm737); Ex[Punc-25::ddr-2]*	23	60	38	0.272[e]
KU1217	*ddr-2(ok574); Ex[Pshc-1::shc-1]*	33	53	62	0.007[b]
KU1219	*svh-2(tm737); Ex[Pshc-1::shc-1]*	15	51	29	1[e]
KU1218	*ddr-2(ok574); Ex[Pshc-1::shc-1(R234K)]*	20	50	40	0.852[b]
KU1220	*ddr-2(ok574); Ex[Pshc-1::shc-1(R136K)]*	38	53	72	<0.001[b]
KU501*	wild type (24 hr after cutting)	43	60	72	-
KU501**	wild type (72 hr after cutting)	46	63	73	1[g]

KU1226*	*ddr-2(tm797)* (24 hr after cutting)	20	69	29	<0.001[a]
KU1226**	*ddr-2(tm797)* (72 hr after cutting)	31	54	57	0.002[h]
KU1206*	*emb-9(g34)* (24 hr after cutting)	19	51	37	-
KU1206**	*emb-9(g34)* (72 hr after cutting)	44	59	75	<0.001[i]
KU503*	*svh-2(tm737)* (24 hr after cutting)	18	51	35	-
KU503**	*svh-2(tm737)* (72 hr after cutting)	25	51	49	0.22[j]

a: vs wild type (KU501), b: vs *ddr-2* (KU1201), c: vs *emb-9* (KU1206), d: vs *mlk-1* (KU504), e: vs *svh-2* (KU503), f: vs *svh-1* (KU502), g: vs wild type (KU501*), h: vs *ddr-2* (KU1226*), i: vs *emb-9* (KU1206*), j: vs *svh-2* (KU503*), k: data from Ref. [7]
