# Supplementary material for: The C. elegans Discoidin Domain Receptor DDR-2 Modulates the Met-like RTK–JNK Signaling Pathway in Axon Regeneration
Source: PLoS Genet. 2016 Dec 16;12(12):e1006475. doi: 10.1371/journal.pgen.1006475 (PMC5161311; doi:10.1371/journal.pgen.1006475)
Supplement: S2 Table — (PDF) [file pgen.1006475.s005.pdf]

S2 Table

KU501	*juIs76 II.*
KU503	*juIs76 II; svh-2 (tm737) X.*
KU504	*juIs76 II; mlk-1(km19) V.*
KU1201	*juIs76 II; ddr-2 (ok574) X.*
KU1202	*juIs76 II; ddr-2 (ok574) X; kmEx1201*
KU1203	*juIs76 II; ddr-2 (ok574) X; kmEx1202*
KU1205	*juIs76 II; ddr-2 (ok574) X; kmEx1204*
KU1206	*juIs76 II; emb-9(g34) III.*
KU1207	*juIs76 II; emb-9(g34) III; ddr-2 (ok574)X.*
KU1208	*juIs76 II; emb-9(g34) III; kmEx1202*
KU1209	*juIs76 II; ddr-2 (ok574) X; kmEx1205*
KU1210	*juIs76 II; mlk-1(km19) V; ddr-2 (ok574) X.*
KU1211	*juIs76 II; ddr-2 (ok574) X; kmEx507*
KU1212	*juIs76 II; mlk-1(km19) V; kmEx1202*
KU1213	*juIs76 II; ddr-2 (ok574) svh-2 (tm737) X.*
KU1214	*juIs76 II; ddr-2 (ok574) X; kmEx1206*
KU1215	*juIs76 II; svh-2 (tm737) X; kmEx1202*
KU1217	*juIs76 II; ddr-2 (ok574) X; kmEx1181*
KU1218	*juIs76 II; ddr-2 (ok574) X; kmEx1207*
KU1219	*juIs76 II; svh-2 (tm737) X; kmEx1181*
KU1220	*juIs76 II; ddr-2 (ok574) X; kmEx1208*
KU1221	*kmEx1209*
KU1222	*kmEx1210*
KU1225	*juIs76 II; ddr-1(ok874) X.*
KU1226	*juIs76 II; ddr-2(tm797) X.*
KU1227	*ddr-2 (ok574) X; kmEx1213*
KU1228	*ddr-1(ok874) ddr-2 (ok574) X.*
KU1229	*juIs76 II; ddr-2(ok574) X; kmEx1209*
KU1230	*juIs76 II; svh-1(ok2531) IV; kmEx1202*
KU1231	*juIs76 II; ddr-2(ok574) X; kmEx505*
KU1232	*juIs76 II; svh-1(ok2531) him-8 (e1489) IV; ddr-2(ok574) X.*
KU1233	*cle-1(cg120) I; juIs76 II.*
KU1234	*juIs76 II; fmi-1(rh308) V.*
KU1235	*juIs76 II; nid-1(cg119) V.*
KU1236	*kmEx1214*

KU1237	*kmEx1215*
KU1238	*kmEx1216*
